# Impact of Stove Renovation on PM_2.5_ Exposure, Risk Perception, Self-Protective Willingness of Rural Residents

**DOI:** 10.3390/toxics11030245

**Published:** 2023-03-05

**Authors:** Lei Huang, Yuxin Liu, Yangyang Wu, Ziwen Ye, Futian Ren, Xinlei Liu, Guofeng Shen

**Affiliations:** 1 State Key Laboratory of Pollution Control & Resource Reuse, School of the Environment, Nanjing University, Nanjing 210023, China; 2 Nanjing University (Suzhou) High-Tech Institute, Suzhou 215123, China; 3 Laboratory for Earth Surface Processes, College of Urban and Environmental Sciences, Peking University, Beijing 100871, China

**Keywords:** stove renovation, PM_2.5_ exposure, risk perception, self-protective willingness

## Abstract

To improve household air quality, the Chinese government has launched a number of pilot stove renovation projects, but few studies have explored the impact of the project on people’s perception of and willingness to participate in these renovations; moreover, factors affecting willingness to pay for the project in rural China are not yet clear. We conducted a field measurement and a corresponding door-to-door questionnaire survey using the renovated group and the unrenovated group. The results showed that (1) the stove renovation project could not only reduce PM_2.5_ exposure and the excess mortality risk of rural residents, but also (2) improve residents’ risk perception and self-protective willingness. (3) Specifically, the project had a deeper impact on female and low-income residents. (4) Meanwhile, the higher the income and the larger family size, the higher the risk perception and self-protective willingness. (5) Furthermore, willingness to pay for the project was related with residents’ support for the project, benefit from renovation, income, and family size. Our results recommended that stove renovation policies should pay more attention to families with lower income and smaller size.

## 1. Introduction

As a significant cause of premature death, household air pollution has also resulted in significant health, economic, and social consequences [1,2]. As the combustion of solid fuels is one of the important sources of household air pollution [3], over the past 30 years, organizations around the world have made many efforts to improve energy for domestic use [4]. The number of deaths due to the household air pollution from solid fuels has continuously declined from 4.4 million in 1990 to 2.3 million in 2019, based on the data from the Global Burden of Disease (GBD). In China, several large-scale stove renovation intervention projects have been carried out, and they has proven effective in reducing household air pollution and the associated burden of disease [5,6,7,8].

However, there is still uneven development in the clean energy renovation movement. In many areas, the implementation of this initiative has not proceeded smoothly due to lagging cognitive levels or economic constraints of the local residents [9,10]. A survey in Beijing discovered that the transition to clean fuels is slower in low-income areas than that in high-income areas [11]. These areas do not currently have the resources for electric or gas heating, by which the old stoves could be converted to clean stoves or replaced with clean coals. In addition, even after stove renovation, there was often a regression from clean to solid fuels when residents perceived solid fuels to be cheaper, more reliable, and safer [12]. Furthermore, rural residents’ risk perception ability and expression ability are generally weaker than those of urban residents [13,14]. In rural areas, long-term failure to convert to clean stoves has serious public health, social, and environmental consequence [15]. Therefore, identifying factors that promote stove renovation for households in low-income areas is critical to achieving sustainable stove renovation for households in the future [16].According to the Third National Agricultural Census in 2016, only 11.9% of villages nationwide and 8.4% in Shanxi province, a major coal mining area, were connected to natural gas. Household solid fuel combustion is still a major source of indoor air pollution in many rural areas of China.

As shown in Table 1, previous studies have considered the impact of clean energy renovation on air quality, individual health and psychological well-being [11,17,18,19,20], while few studies have considered the impact of clean energy intervention on individuals’ perception and willingness to protect themselves. On the other hand, in many developing countries, such as India, it has been found that stove renovation projects were limited by local perceptions and economies [12,21,22,23,24,25,26], while the factors that influence the willingness to pay for stove renovation in rural China are not yet clear. 

Here, we evaluated the implementation effects of the stove renovation project in reducing PM_2.5_ exposure level, as well as improving risk perception and self-protective willingness of rural residents. Moreover, we explored the differences in the impact of stove renovation on different populations, identifying which groups of residents exhibited higher risk perception and willingness to protect themselves. Furthermore, we identified factors that promoted stove renovation. Our findings were important for exploring the prospect of household stove renovation in other rural areas of China, providing a reference for risk communication between the government and the public, and also helping to improve the disparity between urban and rural areas, as well as promoting social equity.

## 2. Materials and Methods

### 2.1. Study Site

**Figure 1:**
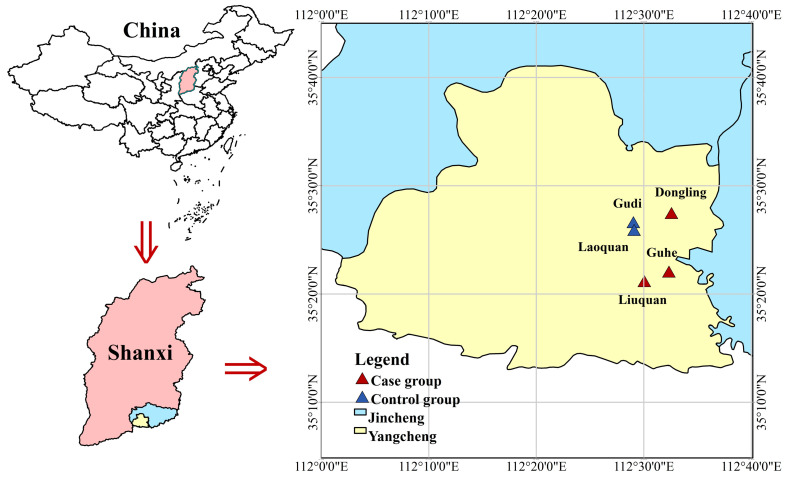
Location of study areas and sampling site.

Located at the southeastern tip of Shanxi Province, Yangcheng County of Jincheng City, one of the 100 key coal-producing counties in China, can be used as a pilot intervention, where coal is the main source of domestic energy for natives [29], and some part of households can be retrofitted with clean cookers using clean coal, while some part cannot. The study area and sampling site of Yangcheng County are depicted in Figure 1.

### 2.2. Field Measurement

We set up one sampling site for each residential household (living room, bedroom, kitchen, etc.) in the above five villages in Yangcheng County. The dates for the stove renovation were from 14 December to 20 December 2019. Our PM sensing instruments were installed from the end of November to December in 2019. The indoor PM_2.5_ concentrations were measured daily in the field by sensing instruments until 31 March 2020, and details of the sensing instruments are listed in Table S1. The mean outdoor PM_2.5_ concentrations of six state-controlled monitoring stations in Jincheng City during the field measurement period (from December, 2019 to March, 2020) are shown in Table S2.

### 2.3. Questionnaire Survey

#### 2.3.1. Sample Selection

Our surveys were carried out during November to December in 2019, with three villages (Guhe, Liuquan, and Dongling) undergoing stove renovation set as the case group and two villages (Laoquan and Gudi) not undergoing stove renovation set as the control group. We went door to door to conduct questionnaire surveys, with a total of 205 questionnaires returned, including 97 for the case group and 108 for the control group; the sample structure is shown in Table S3. The distribution of population characteristics was approximately the same in both groups, and our sample was generally more skewed towards older, less educated, and lower-income groups, since the survey was conducted in rural areas. All respondents were interviewed face-to-face by senior students from the Nanjing University School of the Environment who had been well trained in survey techniques. The research was approved for human subjects by the institutional review board of Nanjing University.

#### 2.3.2. Questionnaire Design

The questionnaire was designed based on psychometric paradigm methods, with minor modifications based on the circumstances of the Chinese residents. Before the formal survey was conducted, we conducted a pre-survey targeting the older age public on a small scale and refined the survey iteratively after the elders provided feedback on a subsequent version. The questionnaire mainly consisted of four sections, as shown in Text S1. The first part included 16 questions to measure health risk perception regarding household air pollution, as well as attitudes towards the stove renovation project. The second part investigated respondents’ willingness to adopt the three main protective behaviors: opening windows, using air purifiers and placing green plants. The response to each question in both parts was ranked on a 5-point Likert-type scale ranging from “1 = minimum” to “5 = maximum”. In the third part, we first described to the respondents the advantages of renovated stoves and clean coal, and then guided the respondents to indicate their willingness to pay for stove renovation and clean coal by means of discrete payment cards. For the case group, we investigated respondents’ willingness to pay for clean coal; while for the control group, we investigated respondents’ willingness to pay for clean coal, as well as stove renovation. The last part of the questionnaire was designed to collect the respondents’ demographic characteristics, including gender, age, education, income, family size, BMI, physical condition and exercise status.

Internal consistency was sufficient for the overall scale (α of case group = 0.81, α of control group = 0.85). Our scale met the requirements of KMO and Bartlett’s Test of Sphericity (Table S4), suggesting that our questionnaire scale was suitable for factor analysis. Maximum Likelihood Estimate was used to process factor downscaling analysis, and confirmatory factor analysis was also performed. The basic indicators GFI, CFI, NFI, and RMSEA met these requirements (Table S5), indicating a good degree of data and model fit. In addition, for a better comparison, willingness to pay by discrete payment cards was divided into four groups: “0~50” = 1, “51~100” = 2, “101~200” = 3, and “>200” = 4.

### 2.4. Data Analysis

#### 2.4.1. Health Risk Assessment

**Figure 2:**
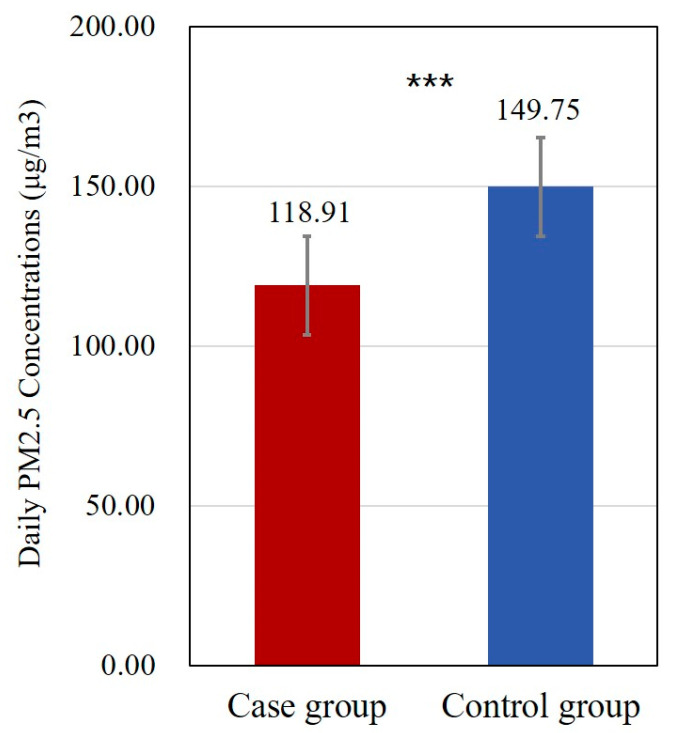
Daily mean PM_2.5_ concentrations. *** *p* < 0.001.

After collecting the data by PM sensing instruments, we eliminated missing values and outliers, and finally obtained the indoor mean PM_2.5_ concentrations in the case group and the control group (Figure 2). An independent sample t test was first used to compare the indoor PM_2.5_ concentrations between the case and the control group, as measured in the field. Furthermore, the daily mean indoor PM_2.5_ concentrations in the case and control groups were used to infer and assess the associated excess risk of mortality in the rural areas of Jincheng City, Shanxi Province, and Northern China under two circumstances: if none of the residents had improved their stoves, and if all residents had improved their stoves. 

According to the health industry standard WS/T 666-2019 of the People’s Republic of China, the health risk assessment model of short-term exposure (Equation (1)) was used to estimate the number of excess deaths caused by PM_2.5_.

(1) $$\Delta y=Pop\times I_{0}\times \left(e^{\beta x_{1}-x_{0}}-1\right)$$

where Δy is total number of excess deaths due to PM_2.5_ exposure (person); Pop is the total resident population in the rural areas of Jincheng City, Shanxi Province, or Northern China at the end of 2019 (person). $I_{0}$ is the rate of mortality of Jincheng City, Shanxi Province, or Northern China in 2019 (‰). The specific data of Pop and $I_{0}$ is shown in Table S6. β is the exposure-response relationship coefficient of PM_2.5_, selected with reference to the results that for every 10 $\mu g/m^{3}$ increase in PM_2.5_ concentration, the risk of total population mortality increased by 0.40% (95% CI: 0.19%; 0.62%), which was studied through meta-analysis of the effect of PM_2.5_ on population mortality in China, conducted by Xie, et al. [30]; $x_{1}$ is the daily mean PM_2.5_ concentrations of the case group or control group measured in the field ($\mu g/m^{3}$); $x_{0}$ is the standard concentration ($\mu g/m^{3}$), for which we chose the WHO PM_2.5_ 24 h mean standard of 25 $\mu g/m^{3}$, and the risk of mortality due to PM_2.5_ exposure above this standard is called the excess risk of mortality. 

#### 2.4.2. Comparison of Risk Perception, Protective Behaviors, and Willingness to Pay

Since our sample did not meet the assumption of a normal distribution, Mann–Whitney U tests were conducted for comparative analysis of risk perception factors, three protective behaviors, and willingness to pay for clean coal between the case group and the control group in order to explore the impact of stove renovation on risk perception and self-protective willingness. 

#### 2.4.3. Impact of Stove Renovation on Different Populations

In order to further analyze the impact of stove renovation on different populations, Mann–Whitney U tests were conducted for comparative analysis of risk perception factors, three protective behaviors, and willingness to pay for clean coal between the case group and the control group under each different demographic characteristics group. 

#### 2.4.4. Variance Analysis among Different Populations

Moreover, Mann–Whitney U tests were used to explain the differences between risk perception factors, protective behaviors, and willingness to pay across different demographic variables.

#### 2.4.5. Multiple Linear Regression Models

Furthermore, 8 multiple linear regression models were used to explore the effects of demographic variables and health risk perception factors on protective behaviors and willingness to pay. For willingness to adopt protective behaviors, including 3 behaviors (opening windows, using air purifiers, and placing green plants) in the case and control groups, respectively, 6 multiple linear regression models were set up to analyze the influence of demographic variables and risk perception factors on protective behaviors. Additionally, we set up 2 models of willingness to pay for stove renovation and clean coal in the control group. Risk perception factors, age, and BMI were defined as continuous variables. Gender was divided into two groups: female = 1, and male = 2. Education level was divided into two groups: primary school and below=1, junior high and above = 2. Monthly income was divided into two ranges (Chinese Yuan, or CNY): “<2000” = 1, “≥2000” = 2. Family size was divided into two groups: “≤” = 1, “>2” = 2. Physical condition was divided into two groups: healthy=1, unhealthy = 2. Exercise status was divided into two groups: non-exerciser = 1, exerciser = 2. 

Multicollinearity tests were performed for multiple linear regression models (Table S7). In addition, the R^2^ statistic of each model was obtained to analyze the explanatory force of the independent variables on the dependent variable, and the F statistic was used to test the credibility of the R^2^ statistic.

All analyses were performed in SPSS 26.0, AMOS 24.0, and ArcMap 10.8.

## 3. Results

### 3.1. Impact of Stove Renovation on PM_2.5_ Exposure

The household daily mean PM_2.5_ concentration, measured in the case and control groups, are shown in Figure 2. Exposure to household PM_2.5_ in the case group was significantly lower than that in the control group (*p* < 0.001), due to the stove renovation.

Based on the above PM_2.5_ levels, the associated excess deaths in the unrenovated group and the renovated group for all causes in the whole rural areas of Jincheng City, Shanxi Province, and even Northern China, respectively, in 2019 are presented in Table 2. It was obvious that stove renovation had made a significant impact on PM_2.5_ exposure, since it reduced household PM_2.5_ pollution, as well as associated excess risk of mortality. 

### 3.2. Impact of Stove Renovation on Risk Perception, Protective Behaviors, and Willingness to Pay

**Figure 3:**
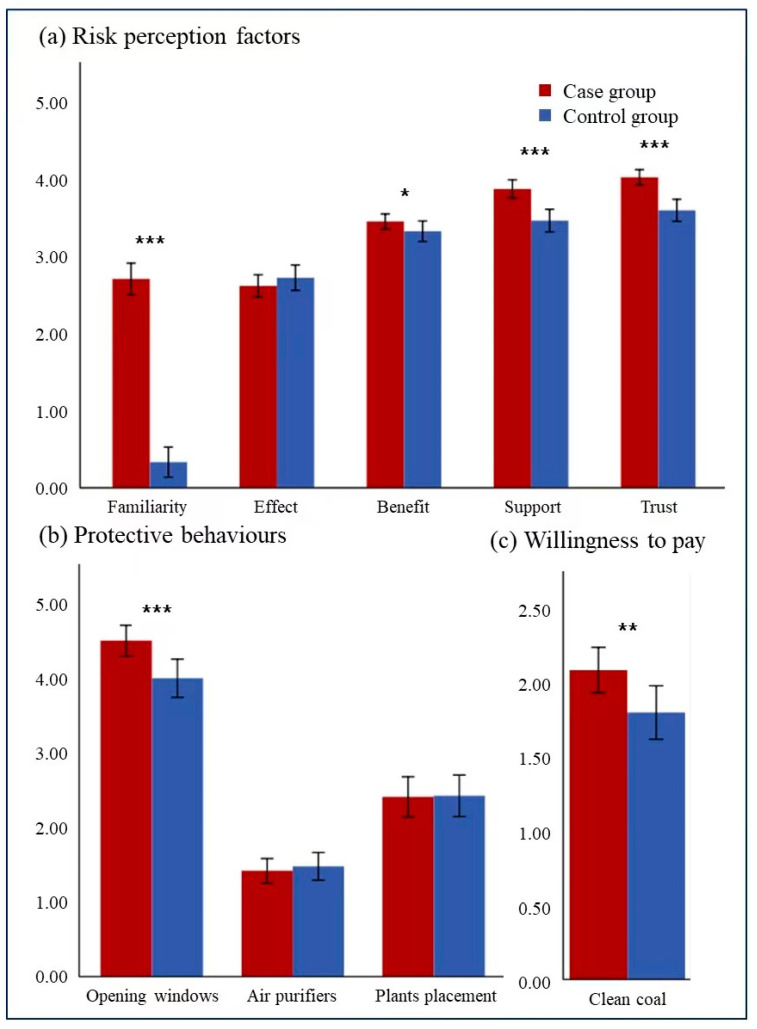
Comparison of (**a**) risk perception factors, (**b**) protective behaviors, and (**c**) willingness to pay between case group and control group. The error bars on the graph represent the standard deviation of the mean. *** *p* < 0.001, ** *p* < 0.01, * *p* < 0.05.

As shown in Figure 3a, significant changes in respondents’ risk perception have taken place between the control and case groups. Perceived familiarity with the stove renovation in the case group was significantly higher than that in the control group (Mean case = 2.71, Mean control = 0.33, *p* = 0.00). Besides, the perceived benefit from the renovation in the case group was significantly higher than that in the control group (mean case = 3.45, mean control = 3.33, *p* = 0.03). Moreover, the respondents’ support for the project in the case group was significantly higher than that in the control group (Mean case = 3.88, Mean control = 3.47, *p* = 0.00). Additionally, respondents’ trust in the government in the case group was significantly higher than that in the control group (mean case = 4.03, mean control = 3.60, *p* = 0.00). However, we did not find any significant difference in the perceived effect between the control and case group (mean case = 2.62, mean control = 2.73, *p* = 0.42).

Secondly, as for the protective behavior scores (Figure 3b), respondents’ protective behavior scores for opening windows in the case group was significantly higher than that in the control group (mean case = 4.49, mean control = 3.99, *p* < 0.001). However, we do not observe any significant differences in the other two protective behaviors between the control and the case groups. Although the scores of using air purifiers in the case group was slightly lower (mean case = 1.41, mean control = 1.47, *p* > 0.05), the scores for placing plants in the case group was slightly lower (mean case = 2.40, mean control = 2.42, *p* > 0.05) as well.

Thirdly, the average amount of money that participants were willing to pay for clean coal in the case group (CNY 115.52) was higher than that of control group (CNY 87.78), as shown in Table S8. After grouping, willingness to pay (Figure 3c) in the case group was significantly higher than that in the control group (mean case = 2.093, mean control = 1.806, *p* = 0.00).

Generally, significant differences could be observed in the public’s risk perception of familiarity, benefit, support, and trust, as well as the willingness to open windows and to pay for clean coal, between the case and the control group, suggesting that stove renovation played an important role in improving people’s risk perception and willingness to protect themselves.

### 3.3. Analysis of the Impact of Stove Renovation on Different Populations

As presented in Table 3, the case group had a significantly higher level of familiarity with the stove renovation project than the control group in all areas. While in all cases, willingness to use air purifiers or place green plants seemed to be unaffected by the project. 

Moreover, whether male or female, with higher or lower education level, higher or lower income level, and whether or not they had a disease, the impact of stove renovation on the respondents’ improvement of risk perception and willingness to protect themselves was significant.

However, for respondents between the ages of 50 and 59, the impact of stove renovation on their increased risk perception and willingness to protect themselves was more significant than for those of other age groups. Moreover, for respondents with a family size of 2 or less, the impact of stove renovation was more significant than that of respondents with a family size of 2 or more.

In addition, for female, lower-income, disease-free respondents, stove renovation increased their willingness to pay for clean coal.

In a word, the implementation of the stove renovation project had a more profound impact on respondents aged 50–59, with smaller family sizes, who were female, had lower incomes, and exhibited better health.

### 3.4. Comparison of Risk Perception and Self-Protective Willingness among Different Populations

As displayed in Table 4, differences when comparing the populations in case and control groups varied widely, while the case and control groups had only one thing in common: respondents with a larger family size were significantly more willing to open windows for ventilation than respondents with a smaller family size.

First, for the comparison of risk perception, the risk perception of male was generally higher than female, which was significant in the case group but not in the control group. Additionally, respondents with higher incomes and larger family sizes were more familiar with the project of stove renovation. Additionally, respondents with large family sizes had a higher level of trust in the government.

Next, for the comparison of protective behaviors, younger respondents were more inclined than older people to open windows for ventilation and use air purifiers. In addition, respondents with higher levels of education, larger family sizes, and better health were more likely to open windows for ventilation. Moreover, respondents with higher incomes were more willing to place green plants.

Finally, for the comparison of willingness to pay for clean coal, respondents with higher incomes and larger family sizes seemed to be more willing to pay for clean coal.

In conclusion, respondents with higher incomes and larger family sizes had a higher risk perception and self-protection awareness and were willing and able to pay for clean air.

### 3.5. Influencing Factors of Protective Behaviors and Willingness to Pay

The socio-economic characteristics and the five factors of risk perception were subjected to regression analysis for protective behaviors, as shown in Table 5, as well as willingness to pay, as shown in Table 6. Variance inflation factors were all much less than 10 (Table S7), so the linear regression models did not exhibit the problem of multicollinearity.

#### 3.5.1. Protective Behaviors

First, model 1 displayed that willingness to open windows in the case group was significantly influenced by support (β = 0.29, *p* = 0.04). R^2^ = 0.23, F = 1.93, *p* = 0.04, indicating that there was a 96% confidence level that the independent variables have a 23% explanatory power for willingness to pay for stove renovation, while model 2 showed that it was significantly affected by income (β = −0.22, *p* = 0.03) and BMI (β = −0.28, *p* = 0.00) in the control group. R^2^ = 0.30, F = 3.04, *p* = 0.00, indicating that there was an over 99% confidence level that independent variables have a 30% explanatory power for willingness to pay for stove renovation.

Next, model 4 demonstrated that willingness to use air purifiers was significantly influenced by familiarity (β = 0.33, *p* = 0.00) in the control group. R^2^ = 0.24, F = 2.32, *p* = 0.01, indicating that there was a 99% confidence level that independent variables have a 24% explanatory power for willingness to pay for stove renovation. The F value of model 3 was not significant. 

Last, model 6 showed that willingness to place green plants in the control group was significantly influenced by physical condition (β = 0.23, *p* = 0.02), familiarity (β = 0.31, *p* = 0.00), and trust (β = 0.32, *p* = 0.02). R^2^ = 0.27, F = 2.67, *p* = 0.00, indicating that there was an over 99% confidence level that independent variables have a 27% explanatory power for willingness to pay for stove renovation. The F value of model 5 was not significant.

Overall, the willingness to residents to protect themselves was significantly influenced by individual differences and the level of risk perception, indicating that residents with higher risk perception were more likely to adopt protective behaviors.

#### 3.5.2. Willingness to Pay

Model 7 showed that income (β = 0.38, *p* = 0.00), BMI (β = 0.19, *p* = 0.03), and support for the project (β = 0.29, *p* = 0.04) played a significant and positive role in willingness to pay for stove renovation. R^2^ = 0.36, F = 4.13, *p* = 0.00, indicating that there was an over 99% confidence level that independent variables have a 36 % explanatory power for willingness to pay for stove renovation.

Model 8 showed that family size (β = 0.42, *p* = 0.00) and benefit from renovation (β = 0.29, *p* = 0.01) played a significant and positive role in the willingness to pay for clean coal. R^2^ = 0.23, F = 2.16, *p* = 0.02, indicating that there was a 98% confidence level that independent variables have a 23% explanatory power for willingness to pay for clean coal.

## 4. Discussion

As far as the stove renovation project itself was concerned, the benefits it generates were positive. The first finding of our study was that the stove renovation could significantly reduce residents’ household exposure levels and lower the associated excess mortality risk. At the same time, economically, lower PM_2.5_ levels produced less social costs [31], effectively alleviating the inequality of health spending [32]. Other studies also confirmed the same results [17,18,19], which again validated the physical utility of stove renovation.

In terms of cognition, stove renovation had also improved the residents’ risk perception and enhanced their self-protective willingness. A study in Malawi reached similar conclusions that when the exposure level decreased, the perceived risk level of the case group residents increased [33]. In addition, an experiment on a heat exposure intervention found that protective behavior scores were higher in the case groups than in the controls [27]. In our study specifically, not only familiarity with the project, benefit from the project, support for project, and trust in government, but also willingness to open windows and to pay for clean coal, were higher in the case group than in the control group; however, a significant difference in effect was not found, with the possible explanation that the original stove and coal had a strong effect on both the case and control groups. Therefore, integrating the physical and conscious functions of stove renovation, we recommend persistently promoting the implementation of stove renovation projects in rural areas. 

Furthermore, by stratifying the population and analyzing the impact of stove renovation under each trait group, we obtained more interesting information. For most groups, both perception and willingness scores in the case group were significantly higher than those in the control group, while for respondents aged 50–59 with smaller family sizes, the impact of stove renovation was more pronounced than for those in other age groups and with larger family sizes, with more significant variables. In addition, for female, lower-income, healthier respondents, stove renovation significantly increased their willingness to pay, which revealed that the project had a positive advocacy effect on relatively disadvantaged groups, such as female residents and those with lower incomes in rural areas. Therefore, more attention should be paid to these groups when implementing stove renovation policies. 

According to our findings, females, the elderly, and residents with lower education levels lacked understanding of the renovation project, self-protective awareness, and willingness. This could be attributed to the lack of sufficient methods and motivation for these groups to understand the relevant policies and health risks [22]. Due to their personal experiences, social expectations, education, and other factors, female and the elderly often lacked awareness and relied more on the opinions of their husbands or children [34,35]. It was also difficult for low-education groups to obtain sufficient information, so they did not have enough cognition about relevant risks [24,36].

In our study, we also found that the willingness to pay for clean coal was positively affected by family size. In larger families, more people were exposed than in smaller families, so respondents with larger family sizes had higher risk perceptions and higher willingness to protect themselves [37,38]. The number of children in a family was positively correlated with self-protection willingness [39]. Considering that in traditional Chinese families, a larger family size often means more susceptible groups, such as the elderly and children, in the family, the adults were often more willing to provide a healthy environment for the elderly and children. When the increase in family size came from the increase in the number of the elderly and children, self-protective willingness increases with family size. On the other hands, our findings were consistent with previous studies [40,41], in which willingness to pay for stove renovation was positively affected by income. The negative impact of giving up the use of clean energy was long-term and uncertain, while the negative impact of bearing economic pressure was urgent and definite. In lower-income rural areas, the government could consider increasing subsidies in less economically developed areas when implementing stove renovation subsidy policies [11]. Priority can be given to households with higher income and larger family sizes before gradually extending the implementation to other households, improving residents’ cooperation, reducing resistance to the project promotion, and effectively reducing PM_2.5_ exposure levels. 

Apart from family size and income level, the willingness to protect and pay was indeed facilitated by risk perception, as found in previous studies [42,43,44]. Moreover, risk perception was a positive indicator of population adaptive behavior [45,46]. The government and the media should pay more attention to household air pollution and strengthen risk communication, which would also help to enhance the public’s trust in the government and household air pollution governance.

However, many cases found that due to factors such as lack of awareness and economic constraints, residents in many areas still tended to use traditional stoves after stove renovations [22,25]. It should be noted that stove renovation was not a single, one-time project occurring in one location until the renovation was completed, but rather a long-term systematic project, progressing from the early publicity stage to later tracking. After the renovation was completed, continuous tracking and continuous improvement by the residents were constantly required. Corresponding subsidies were used to not only support residents in replacing stoves, but also to support them in continuing to use new stoves for a long period time, maintaining residents’ long-term dependence on clean energy [34,35].

## 5. Conclusions

This study demonstrated that stove renovation could effectively reduce PM_2.5_ exposure level and the risk of excess mortality, as well as improve residents’ risk perception and self-protection awareness. Moreover, the project had a positive advocacy effect on relatively disadvantaged groups, such as females and residents with lower incomes in rural areas. We confirmed the facilitative effect of risk perception on protective behaviors. Lastly, we discovered that the stove renovation project could be initiated with residents with higher incomes and larger family sizes. The results can help policy makers better understand the factors that influence sustained adoption of clean cooking systems in rural areas. The findings of this study contribute, to some extent, to environmental equity and help increase risk perception and self-protective awareness among residents in rural areas by promoting risk communication.

However, our study has the following limitations and follow-up prospects: (1) The sample size of this study was slightly inadequate due to time and effort constraints, and follow-up studies may consider expanding the sample size. (2) This study did not match the pollutant concentrations of each household with individual risk perception, and subsequent studies could analyze the relationship between pollutant concentrations and individual characteristics, risk perception, and self-protective willingness in more depth by matching the pollutant concentrations of each household. (3) This study only used controls for the case and control groups at the same time, but lacked longitudinal controls at different times; the follow-up study should consider conducting long-term longitudinal follow-up surveys on the sample population, measuring and conducting comparative analysis after the intervention project of stove renovation had been completed for a period of time, in order to further explore the changes in residents’ risk perception levels under the intervention measures.

## Figures and Tables

**Table 1:** Research gaps.

Regions	References	Main Contents	Limitations and Inspiration
Impact of clean energy interventions.			
Beijing, China	Barrington-Leigh C et al., 2019 [11]	The promoting impact of clean heating renovation on indoor air quality and people’s well-being.	The impact of the project on health was not quantified.
Shanxi Province, China	Zhao B et al., 2021 [17]	The promoting impact of clean heating renovation on people’s health.	The impact of interventions on people’s perception and self-protective willingness was not considered.
North China	Meng W et al., 2022 [18]	The synergistic impact of air pollution control action plan on people’s physiology and psychology.	
North China	Nan Zhao et al., 2022 [19]	The promoting impact of air pollution control action plan on the environment, health, and the economy.	
China	Meng W et al., 2021 [20]	The promoting impact of interaction between stove renovation and energy conversion on environment and health.	
Jiangsu Province, China	Lou J et al., 2021 [27]	The promoting impact of high temperature interventions on people’s risk perception and protective willingness.	The impact of interventions targeting air pollution on perception and willingness was not considered.
Factors affecting willingness to pay in rural China.			
India	Smitha Rao et al., 2020 [21]	Rural women had insufficient awareness and knowledge of the health risks associated with traditional stoves.	In countries such as India, stove renovation projects were limited by residents’ perception and economic conditions. These limitations were not considered for rural China.
India	Nishesh Chalise et al., 2018 [22]	Clean cooking techniques were difficult to sustain.	
India	Gould CF et al., 2020 [23]	Education and attitudes played a role in the choice of cooking fuel.	
Peru	Hollada J et al., 2017 [24]	Lack of awareness and attention to health risks was one of the barriers to continued use of clean stoves.	
Chile	Boso À et al., 2019 [25]	Risk perception influenced people’s willingness to renovate stoves.	
Rwanda	Campbell CA et al., 2021 [26]	Stove renovation was limited by residents’ awareness.	
Nigeria	Jewitt S et al., 2020 [12]	Stove renovation was limited by residents’ economic conditions.	
Beijing, China	Yana Jin et al., 2020 [28]	The willingness to pay for clean air was positively influenced by income levels.	Other influences related to the willingness to pay for clean air in rural China were not considered.

**Table 2:** Number of excess deaths (person) ^1^.

	Unrenovated Group	Renovated Group	Benefit ^2^
Rural areas of Jincheng City	251	188	63
Rural areas of Shanxi Province	4515	3378	1137
Rural areas in Northern China	18,794	14,061	4733

^1^ Note: excess deaths in areas of Jincheng City, Shanxi Province, and the whole area of Northern China are based on the measured PM_2.5_ concentrations of the control and case group. ^2^ Note: the formula for yielding “Benefit” was: excess deaths of Unrenovated group-excess deaths of Renovated group.

**Table 3:** Increase ratio in case group compared to control group ^1^.

Variables	Sort	Familiarity (‰)	Benefit (%)	Support (%)	Trust (%)	Windows ^3^ (%)	Purifiers (%)	Plants ^4^ (%)	Clean coal ^5^ (%)
Gender	Female	149.06 ***	7.80 *	12.82 **	8.81 *	11.46	−8.39	−8.14	19.06 *
	Male	52.24 ***	0.84	11.89 **	15.31 ***	14.20 **	−0.62	6.46	14.13
Age	<50	62.86 ***	1.03	10.85	10.13	13.01 *	7.84	1.22	6.90
	50–59	94.15 ***	10.15 **	19.65 ***	14.69 **	16.13 **	−10.50	2.19	24.57 *
	>=60	58.01 ***	0.20	5.61	11.74	10.05	−12.79	−5.92	16.26
Education ^2^	Lower	85.74 ***	7.78 *	12.11 **	13.39 **	23.54 **	−6.91	5.98	18.18 *
	Higher	67.24 ***	1.57	12.65 **	11.97 **	7.86 *	1.47	−2.16	16.42 *
Family size	<=2	141.69***	2.54	9.10 *	14.53 **	11.92 *	2.06	4.49	32.48 **
	>2	38.68 ***	4.99	11.35 *	5.55	4.85	−13.48	−10.26	−3.43
Income	<2000	97.46 ***	3.77	9.65 **	10.85 **	8.25 *	−6.11	0.97	14.07 *
	>=2000	49.99 ***	3.73	15.57 **	13.80 **	20.45 **	−0.85	−3.27	18.18
Physical condition	Healthy	59.48 ***	5.80 *	14.46 **	10.82 **	15.24 ***	−6.26	0.10	15.94 *
	Unhealthy	111.65 ***	0.44	7.52 *	14.41 **	9.70	−0.82	−1.49	16.06

^1^ Note: the calculation formula for each value of increase ratio in Table 3 was: (Scores of case group-Scores of control group)/Scores of control group × 100% and 1000‰. The original scores for the case and control group are shown in Table S9. ^2^ Note: “Lower” education represents “Primary school and below”, and “Higher” represents “Junior high and above”. ^3^ Note: “Windows” represents “Opening windows”, also in Table 4. ^4^ Note: “Plants” represents “Plants placement”, also in Table 4. ^5^ Note: “Clean coal” represents “Willingness to pay for clean coal”, also in Table 4. *** *p* < 0.001, ** *p* < 0.01, * *p* < 0.05.

**Table 4:** Comparison of risk perception and self-protective willingness among different demographic groups ^1^.

Variables	Familiarity	Benefit	Support	Trust	Windows	Purifiers	Plants	Clean Coal
Case group (%)								
Gender	18.96 *	0.16	5.13 *	5.70 *	3.97	−2.80	0.87	3.48
Age	−13.69	−6.43	−8.73	−4.12	3.18	54.72 *	21.54	−2.54
	6.67	0.91	−0.19	1.83	15.91 **	59.48 **	37.53	8.36
Education	14.55	−0.56	5.08	4.48	2.58	30.46	9.95	8.82
Family size	6.10	1.14	7.24	5.31	16.88 *	4.67	7.96	4.95
Income	23.46 *	4.34	4.78	0.74	7.25	10.24	27.40	19.61 *
Physical condition	4.29	1.28	2.06	2.24	14.85 **	−5.63	3.05	1.28
Control group (%)								
Gender	204.00	7.07	6.00	−0.27	1.47	−10.40	−12.96	7.95
Age	23.37	2.02	−1.49	−0.15	6.03	28.41	22.71	13.58
	−0.43	0.08	−4.90	3.33	12.88	28.97	27.82	17.86
Education	41.99	5.53	4.58	5.80	17.48 *	19.69	19.10	10.47
Family size	230.58 *	−1.22	5.08	14.27 ***	24.76 ***	23.47	25.71	43.97 ***
Income	121.15	4.38	−0.59	−1.87	−3.61	4.38	32.99 *	15.45
Physical condition	4.29	1.28	2.06	2.24	14.85	−5.63	3.05	1.28

^1^ Note: the calculation formula for each value in Table 4 is (in order of each row, from top to bottom): (Scores of male-Scores of female)/Scores of female×100%; (Scores of “<50”-Scores of “50–59”)/Scores of “50–59” × 100%; (Scores of “<50”-Scores of “>=60”)/Scores of “>=60” ×100%; (Scores of “Higher”-Scores of “Lower”)/Scores of “Lower” ×100%; (Scores of “>2”-Scores of “<=2”)/Scores of “<=2” ×100%; (Scores of “>=2000”-Scores of “<2000”)/Scores of “<2000” ×100%; (Scores of “Healthy”-Scores of “Unhealthy”)/Scores of “Unhealthy” ×100%. The original scores are shown in Table S9. *** *p* < 0.001, ** *p* < 0.01, * *p* < 0.05.

**Table 5:** Multiple linear regression models of protective behaviors by demographic variables and risk perception factors.

	Opening Windows		Air Purifiers		Plants Placement	
	Case	Control	Case	Control	Case	Control
Variables	Model 1	Model 2	Model 3	Model 4	Model 5	Model 6
Gender	0.06	0.06	0.10	−0.11	0.07	−0.19
Age	−0.25	−0.21	−0.38 **	−0.12	−0.20	−0.09
Education	−0.15	0.18	0.16	0.03	0.00 ^1^	0.02
Family size	0.03	0.10	−0.11	0.01	0.01	−0.10
Income	−0.07	−0.22 *	−0.06	−0.06	0.09	0.20
BMI	0.02	−0.28 **	−0.08	0.17	−0.06	−0.12
Physical condition	−0.17	0.03	0.23 *	0.12	0.09	0.23 *
Exercise	0.09	−0.01	0.04	0.12	0.09	0.06
Familiarity	0.18	0.13	−0.29 *	0.33 **	−0.08	0.31 **
Effect	0.00	−0.05	0.06	−0.06	−0.05	−0.16
Benefit	0.05	−0.11	0.07	0.07	0.10	−0.07
Support	0.29 *	0.03	0.10	−0.13	0.14	−0.24
Trust	−0.13	0.16	−0.08	0.25	−0.13	0.32 *
R^2^	0.23	0.30	0.21	0.24	0.11	0.27
F	1.93 *	3.04 **	1.65	2.32 *	0.82	2.67 **

^1^ Note: since two significant figures were retained, the result is “0.00”, while the original value was “0.003”. There were several similar cases in Table 5 and Table 6 which were not repeated. *** *p* < 0.001, ** *p* < 0.01, * *p* < 0.05.

**Table 6:** Multiple linear regression models of protective behaviors by demographic variables and risk perception factors.

	Willingness to Pay (Control Group)	
	Stove renovation	Clean coal
Variables	Model 7	Model 8
Gender	0.05	−0.00
Age	0.05	0.17
Education	0.09	0.01
Family size	−0.03	0.42 ***
Income	0.38 ***	0.08
BMI	0.19 *	−0.03
Physical condition	0.03	−0.06
Exercise	−0.11	0.02
Familiarity	−0.03	0.06
Effect	0.17	0.02
Benefit	0.09	0.29 *
Support	0.29 *	0.03
Trust	0.06	−0.08
R^2^	0.36	0.23
F	4.13 ***	2.16 *

*** *p* < 0.001, ** *p* < 0.01, * *p* < 0.05.

## Data Availability

All relevant data are within the manuscript and its Supplementary Information files. The data that support the findings of this study are available upon request from the corresponding author, by sending email requests to Lei Huang; Email: huanglei@nju.edu.cn.

## Supplementary Materials

The following supporting information can be downloaded at: https://www.mdpi.com/article/10.3390/toxics11030245/s1, Text S1: Questionnaire on stove renovation; Table S1: Instrument and tested parameter; Table S2: Monthly mean outdoor PM_2.5_ concentrations at 6 stations in Jincheng City, Shanxi Province; Table S3: Sample structure; Table S4: KMO and Bartlett’s Test of Sphericity; Table S5: Confirmatory factor analysis; Table S6: Population data in Equation (1); Table S7: Variance inflation factors of Models 1~8; Table S8: Description of willingness to pay; Table S9: The original scores of case and control groups.
