# Supplementary material for: Impact of Stove Renovation on PM2.5 Exposure, Risk Perception, Self-Protective Willingness of Rural Residents
Source: Toxics. 2023 Mar 5;11(3):245. doi: 10.3390/toxics11030245 (PMC10051283; doi:10.3390/toxics11030245)
Supplement: Supplementary file 1 [file toxics-11-00245-s001.zip › toxics-2223954-supplementary.pdf]

Supplementary Information

Impact of Stove Renovation on $PM_{2.5}$ Exposure, Risk Perception, Protective Behavior, and Willingness to Pay of Rural Residents

Lei Huang[1,2,*], Yuxin Liu[1,2], Yangyang Wu[1,2], Ziwen Ye[1,2], Futian Ren[1,2], Xinlei Liu[3], Guofeng Shen[3]

[1] State Key Laboratory of Pollution Control & Resource Reuse, School of the Environment, Nanjing University, Nanjing 210023, China

[2] Nanjing University (Suzhou) High-tech Institute, Suzhou 215123, China

[3] Laboratory for Earth Surface Processes, College of Urban and Environmental Sciences, Peking University, Beijing 100871, China

[*] Corresponding author: Lei Huang; Email: huanglei@nju.edu.cn.

Table of Content

Text S1. Questionnaire on the stove renovation.

Table S1. Instrument and tested parameter.

Table S2. Monthly mean outdoor $PM_{2.5}$ concentrations at 6 stations in Jincheng City, Shanxi Province.

Table S3. Sample structure.

Table S4. KMO and Bartlett’s test of sphericity.

Table S5. Confirmatory factor analysis.

Table S6. Population data in Equation 1.

Table S7. Variance inflation factors of Model1~8.

Table S8. Description of willingness to pay.

Table S9. The original scores of the case and control group.

Text S1:

Questionnaire on the stove renovation

Part 1 Perceptions related to stove renovation projects

(1). How much do you know about the content of the project of stove renovation in your village?

1 2 3 4 5

Not at all Not much Average A lot Very much

(2). How much do you know about the difference between the new replacement stove and the original stove (daily coal consumption, smoke emission method, smoke emission)?

1 2 3 4 5

Not at all Not much Average A lot Very much

(3). How much do you know about the difference between bulk coal and clean coal?

1 2 3 4 5

Not at all Not much Average A lot Very much

(4). How often did you learn about the stove renovation project through the following sources?

Please check the box for the understanding method:

☐ Acquaintance communication ☐Telephone ☐ Television ☐ Newspaper ☐ Broadcast

1 2 3 4 5

Never Occasionally Average Often Almost daily

(5). How was the indoor air environment in your home before the stove renovation (was it pungent)?

1 2 3 4 5

Very bad Fairly bad Average Fairly good Very good

(6). Did you experience discomfort when heating or cooking in the kitchen before the stove rennovation?

1 2 3 4 5

Very uncomfortable Fairly uncomfortable Average Slightly uncomfortable No uncomfortable

(7). Do you think the original stove and coal had any effect on your health?

1 2 3 4 5

Not at all Fairly unaffected Average Partially affected Very affected

(8). Are you worried that the stove will cause inconvenience to your daily life during the renovation process?

1 2 3 4 5

Very worried Fairly worried Average Fairly unworried Unworried

(9). How has the stove renovation project made an overall difference in your family's quality of life in your opinion?

1 2 3 4 5

Much worse Fairly worse Average Fairly better Much better

(10). How much impact do you think that the stove renovation project has in your household air quality?

1 2 3 4 5

Not at all Not much Average A lot Very much

(11). How much do you support the stove renovation project?

1 2 3 4 5

Not at all Fairly unsupported Average Fairly supported Very supported

(12). How necessary do you think it is for the government to implement a stove renovation project?

1 2 3 4 5

Not at all Basically unnecessary Average Fairly necessary Very necessary

(13). How likely do you think the stove renovation project is to be rolled out?

1 2 3 4 5

Not at all Basically unlikely Average Fairly likely Very likely

(14). Do you think the stove renovation project can be implemented in accordance with the program?

1 2 3 4 5

Not at all Basically impossible Average Basically possible Completely possible

(15). Do you believe that the government can give a good treatment of household air pollution?

1 2 3 4 5

Not at all Basically impossible Average Basically possible Completely possible

(16). Do you believe the government can guarantee our residents access to cleaner and cheaper energy?

1 2 3 4 5

Not at all Basically impossible Average Basically possible Completely possible

Part 2 Protective behaviours related to household air pollution

Below are some specific measures to alleviate health problems arising from household air pollution, please indicate whether you will take such action in the future (If you have already taken similar action and intend to continue to do so, please select "likely" or "very likely" as a response). Please mark the corresponding option with a tick. (1=very unlikely 2=unlikely 3=uncertain 4=likely 5=very likely)

	Items	1	2	3	4	5
1	Opening windows					
2	Use or purchase air purifiers					
3	Placing green plants					

Part 3 Willingness to pay for the stove renovation and clean coal

Case group:

The stove renovation project can reduce harmful gas emissions, which has great health benefits, and clean coal burns more efficiently. At present, the stove purchase cost of the stove renovation project is all borne by the government, and the maximum subsidy for clean coal is 2.5 tons, and you need to purchase it by yourself later. What is the maximum amount you are willing to pay for clean coal each month?

0	5	10	20	30
40	50	60	70	80
90	100	125	150	175
200	250	300	350	______

Control group:

The stove renovation project can reduce harmful gas emissions and produce great health benefits. The cost for the government to promote this project is about 2,000-3,000 RMB/household and may be

partially subsidized. What is the maximum amount you are willing to pay for stove purchase?

0	50	100	200	500	1000	1500	2000	3000

Clean coal is better quality than the original bulk coal, produces less harmful gases from burning, and burns more efficiently. What is the maximum amount you are willing to pay for clean coal each month?

0	1	2	5	8
10	20	50	80	100
200	300	400	500	600
700	800	900	1000	_____

Part 4 Basic information of respondents

1. Gender：☐Male ☐Female

2. Name：________

3. Tel：______________________

4. Age：_______

5. Height：_______(cm)，Weight：______(kg)

6. Marital status：☐Unmarried ☐Married ☐Divorced ☐ Widowed

7. How many members in your family?_______(Permanent population)

8. Which is your mode of residence? (You can choose more than one option.)
☐ Live alone ☐ Live with spouse ☐ Live with parents ☐ Live with children ☐Others__

9. Your highest level of education: ☐ Uneducated ☐ Primary ☐ Junior high ☐ High/Vocational high ☐ University/College ☐ Graduate student and above

10. Your monthly per capita household income: (Permanent population) ☐None ☐<500yuan ☐500-999yuan ☐1000-1999yuan ☐2000-2999yuan ☐3000-3999yuan ☐4000-4999yuan ☐>5000yuan

11. Occupation of family members: (Permanent population; You can choose more than one option.)
☐Student ☐worker ☐ Retiree ☐ Unemployed or laid-off workers ☐ Professional technicians (Doctor, teacher et al.) ☐ Administrators (village councils) ☐ Commercial, service personnel ☐Farmer ☐ Housewife ☐Others________________

12 How do you think your health is?

1	2	3	4	5
Very poor	Fairly poor	Average	Fairly healthy	Very healthy

13 Do you have social security? ☐Yes ☐No
Do you have other health insurance? ☐Yes ☐No

14 Have you been diagnosed by a hospital with any of the following health conditions? (You can choose more than one option.)
☐Diabetes ☐Hypertension ☐High blood pressure ☐High cholesterol ☐Coronary heart disease ☐Angina pectoris ☐Myocardial infarction ☐Stroke ☐Other cardiovascular disease ☐Emphysema ☐Chronic bronchitis ☐Asthma ☐Other respiratory disease ☐Acute renal failure ☐Pharyngitis ☐Cancer _________ ☐I have never been diagnosed with these problems.

15 Have you ever been hospitalized or seen an emergency room for respiratory diseases such as bronchitis/bronchitis, laryngitis, lung cancer, etc.? ☐Yes ☐No

16 What is your drinking status?
☐Never ☐1-3times/month ☐4-6times/month ☐2-3times/week ☐almost daily

17 Do you smoke?
☐Yes,____cigarette(s) per day;

☐No, are there colleagues or family members around you who smoke regularly?
Your relationship with a smoker is:________; he (she) has smoked________(year)， ________(cigarette /day).

18 How often do you exercise?

☐Never ☐1-3times/month ☐4-6times/month ☐2-3times/week ☐almost daily

Exercises were performed by________.

Table S1. Instrument and tested parameter.

Parameter	Instrument	Resolution	Measuring range	Uncertainty
$PM_{2.5}$	Plantower PMS7003	1 μg/m^3	≥1000 μg/m^3	±10 μg/m^3@0~100μg/m^3<br>±10%@100~500 μg/m^3<br>±10%~±20%@>500 μg/m^3

Table S2. Monthly mean outdoor $PM_{2.5}$ concentrations at 6 stations in Jincheng City, Shanxi Province. (Unit: μg/m^3)

Month	Station1[1]	Station2	Station3	Station4	Station5	Station6	Mean
2019-12	64.797	61.150	62.342	64.070	58.914	44.992	59.378
2020-01	115.392	108.889	112.135	115.118	104.662	96.524	108.787
2020-02	51.522	49.339	49.345	50.841	49.778	42.548	48.896
2020-03	42.200	39.465	38.756	39.302	39.011	30.587	38.220
Mean	68.478	64.711	65.644	67.333	63.091	53.663	63.820

[1] Note："Station1" is "The Water Company", "Station2" is "Baiyun Trade", "Station3" is "Municipal Environmental Protection Bureau", "Station4" is "Technical College", "Station5" is "Zezhou No.1 Middle School", and "Station6" is "White Horse Temple". The above stations are all state-controlled stations in Jincheng City, Shanxi Province.

Table S3. Sample structure.

		Case group (N=97)		Control group (N=108)	
Variables	Sort	N	Percent (%)	N	Percent (%)
Gender	Female	51	52.6	48	44.4
	Male	46	47.4	60	55.6
Age	<50	29	29.9	29	26.9
	50-59	31	32.0	46	42.6
	>=60	37	38.1	33	30.6
Education	Primary school and below	46	47.4	39	36.1
	Junior high and above	51	52.6	69	63.9
Income	<2000	61	62.9	69	63.9
	>=2000	36	37.1	39	36.1
Family size	<=2	35	36.1	64	59.3
	>2	62	63.9	44	40.7
BMI	<23	58	59.8	44	40.7
	>=23	39	40.2	64	59.3
Physical condition	Healthy	58	59.8	70	64.8
	Unhealthy	39	40.2	38	35.2
Exercise	Non-exerciser	64	66.0	92	85.2
	Exerciser	33	34.0	16	14.8

Table S4. KMO and Bartlett's test of sphericity.

		Case group	Control group
KMO		0.81	0.78
Bartlett's Test of Sphericity	Approximate chi-square	750.22	1746.92
	Degree of freedom	120	120
	Significance	0.00	0.00

Table S5. Confirmatory factor analysis.

	GFI	CFI	NFI	RMSEA
Case group	0.851 >0.80	0.928 >0.90	0.823 >0.80	0.074 <0.08
Control group	0.862 >0.80	0.967 >0.90	0.918 >0.90	0.076 <0.08

Table S6. Population data in Equation 1[1].

Areas		Pop (million)	I_0 (‰)
Rural areas of Jincheng City		0.92	5.30
Rural areas of Shanxi Province		15.08	5.85
Rural areas in north China region	Beijing	2.89	5.49
	Tianjin	2.58	5.30
	Hebei Province	32.17	6.12
	Shanxi Province	15.08	5.85
	Inner Mongolia Municipality	9.30	5.66

[1]Note: the data in the table all came from *National Bureau of Statistics of China.*

Table S7. Variance inflation factors of Model1~8.

Variables	Case group	Control group
Gender	1.175	1.346
Age	2.109	1.918
Education	1.397	1.273
Family size	1.332	1.365
Income	1.482	1.351
BMI	1.105	1.155
Physical condition	1.311	1.325
Exercise	1.394	1.202
Familiarity	1.711	1.204
Effect	1.155	1.216
Benefit	1.297	1.591
Support	2.210	2.983
Trust	2.227	2.305

Table S8. Description of willingness to pay.

	N	Minimum	Maximum	Average	Standard Deviation
Case group-clean coal	97	0	700	115.52	120.864
Control group-clean coal	108	0	500	87.78	98.347
Control group-kitchen range	108	0	3000	433.33	597.698

Table S9. The original scores of the case and control group.

Variables	Sort	Group[1]	Familiarity	Benefit	Support	Trust	Windows[3]	Air purifiers	Plants[4]	Clean coal[5]
Gender	Female	1	2.49	3.45	3.78	3.92	4.41	1.43	2.39	2.06
		2	0.16	3.20	3.35	3.60	3.96	1.56	2.60	1.73
	Male	1	2.96	3.46	3.98	4.14	4.59	1.39	2.41	2.13
		2	0.48	3.43	3.56	3.59	4.02	1.40	2.27	1.87
Age	<50	1	2.64	3.39	3.76	4.00	4.79	1.90	2.86	2.14
		2	0.36	3.36	3.39	3.63	4.24	1.76	2.83	2.00
	50-59	1	3.06	3.62	4.12	4.17	4.65	1.23	2.35	2.19
		2	0.29	3.29	3.44	3.64	4.00	1.37	2.30	1.76
	≥60	1	2.47	3.36	3.77	3.93	4.14	1.19	2.08	1.97
		2	0.36	3.35	3.57	3.52	3.76	1.36	2.21	1.70
Education[2]	Lower	1	2.52	3.46	3.78	3.93	4.43	1.22	2.28	2.00
		2	0.26	3.21	3.37	3.47	3.59	1.31	2.15	1.69
	Higher	1	2.88	3.44	3.97	4.11	4.55	1.59	2.51	2.18
		2	0.37	3.39	3.52	3.67	4.22	1.57	2.57	1.87
Family size	≤2	1	2.61	3.43	3.70	3.90	4.06	1.37	2.29	2.03
		2	0.17	3.34	3.40	3.40	3.63	1.34	2.19	1.53
	>2	1	2.77	3.47	3.97	4.10	4.74	1.44	2.47	2.13
		2	0.57	3.30	3.57	3.89	4.52	1.66	2.75	2.20
Income	<2000	1	2.49	3.40	3.81	4.02	4.38	1.36	2.18	1.95
		2	0.23	3.28	3.47	3.62	4.04	1.45	2.16	1.71
	≥2000	1	3.08	3.55	3.99	4.05	4.69	1.50	2.78	2.33
		2	0.51	3.42	3.45	3.56	3.90	1.51	2.87	1.97
Physical condition	Healthy	1	2.75	3.47	3.91	4.06	4.74	1.38	2.43	2.10
		2	0.40	3.28	3.41	3.67	4.11	1.47	2.43	1.81
	Unhealthy	1	2.64	3.43	3.83	3.97	4.13	1.46	2.36	2.08
		2	0.22	3.41	3.56	3.47	3.76	1.47	2.39	1.79

[1] Note: "1" of group represents "case group", and "2" of group represents "control group".
[2] Note: "Lower" of education represents "Primary school and below", and "Higher" represents "Junior high and above".
[3] Note: "Windows" represents "Opening windows".
[4] Note: "Plants" represents "Plants placement".
